# Recommendations for selection of target parameters and process recommendations for audiological and technical functional testing of cochlear implant: Prepared by the ERA consortium (AG-ERA) of ADANO in cooperation with the Implantable Hearing Systems expert committee of the DGA. Confirmed by the board of ADANO on 31.01.2025

**DOI:** 10.1007/s00106-025-01629-w

**Published:** 2025-06-05

**Authors:** A. Müller, M. Blümer, O. C. Dziemba, A. Elsholz, L. Fröhlich, U. Hoppe, D. Polterauer, T. Rahne, T. Steffens, M. Walger, T. Weißgerber, T. Wesarg, S. Zirn, T. Rader

**Affiliations:** 1 Hearing Center Berlin (HZB), ORL Department, Head, Neck, Plastic and Cosmetic Surgery, Center for Hearing Implants, Vivantes Klinikum im Friedrichshain, Landsberger Allee 49, 10249 Berlin, Germany; 2 Clinic and Polyclinic for Ear, Nose and Throat Medicine, University Medical Center Hamburg-Eppendorf, Hamburg, Germany; 3 Department of ENT, Head & Neck Surgery, University Medicine Greifswald, Greifswald, Germany; 4 Department of Otolaryngology, Head and Neck Surgery, University Medical Center Bonn, Bonn, Germany; 5 Ear, Nose and Throat Clinic, Head and Neck Surgery, University Hospital Erlangen, Erlangen, Germany; 6 Department of Otorhinolaryngology, LMU Klinikum, Munich, Germany; 7 Department of Otorhinolaryngology, Head and Neck Surgery, University Medicine Halle, Halle (Saale), Germany; 8 Clinic and Polyclinic for Ear, Nose and Throat Medicine, University Hospital Regensburg, Regensburg, Germany; 9 Department of Otorhinolaryngology, Head and Neck Surgery, University of Cologne, Cologne, Germany; 10 Clinic for Ear, Nose and Throat Medicine, University Medicine, Goethe University Frankfurt am Main, Frankfurt am Main, Germany; 11 Department of Otorhinolaryngology, Medical Center—University of Freiburg, Faculty of Medicine, University of Freiburg, Freiburg, Germany; 12 Peter Osypka Institute of Medical Engineering, University of Applied Sciences, Offenburg, Germany

**Keywords:** Quality assurance, Minimum standard, Measurement methods, Evoked potentials, Impedances, Qualitätssicherung, Minimalstandard, Messverfahren, Evozierte Potentiale, Impedanzen

## Abstract

Continuous monitoring of the technical and physiological function of cochlear implants (CI) is a central part of the care process. Despite worldwide efforts to standardise procedures, there is still considerable variation between CI centres, particularly in terms of the methods used, their practical implementation and the definition of meaningful target parameters. A standardised structured test procedure is needed for reliable quality assurance and better comparability. Against this background, the ADANO Working Group for Evoked Response Audiometry (AG-ERA), in close cooperation with the Cochlear Implants and Implantable Hearing Systems Committee of the German Society of Audiology (DGA), developed a minimum standard for audiological and technical functional testing of CIs in an open consensus process. This standard defines basic requirements for performance and documentation and serves as a practical recommendation for CI centres. It is intended to improve interdisciplinary cooperation, increase the quality of care and enable structured long-term optimised care for CI patients.

## Abstract (de)

Die kontinuierliche Kontrolle der technischen und physiologischen Funktion von Cochlea-Implantaten (CI) stellt einen zentralen Baustein im gesamten Versorgungsprozess dar. Trotz weltweiter Bestrebungen zur Vereinheitlichung der Verfahren zeigen sich nach wie vor erhebliche Unterschiede zwischen den CI-versorgenden Einrichtungen – insbesondere hinsichtlich der eingesetzten Methoden, ihrer praktischen Umsetzung und der Festlegung aussagekräftiger Zielgrößen. Für eine verlässliche Qualitätssicherung und verbesserte Vergleichbarkeit ist ein einheitlicher, strukturierter Prüfprozess erforderlich. Vor diesem Hintergrund wurde in einem offenen Konsensverfahren der Arbeitsgruppe Elektrische Reaktionsaudiometrie (AG-ERA) der ADANO, gemeinsam mit dem Fachausschuss „Cochlea-Implantate und implantierbare Hörsysteme“ der DGA, ein Minimalstandard für die audiologisch-technische Funktionsprüfung von CI entwickelt. Dieser definiert grundlegende Anforderungen an Durchführung und Dokumentation und dient als praxisnahe Empfehlung für CI-versorgende Einrichtungen. Ziel ist eine standardisierte, nachvollziehbare Vorgehensweise, die die interdisziplinäre Zusammenarbeit verbessert, die Versorgungsqualität erhöht und eine strukturierte, langfristig optimierte Betreuung von CI-Tragenden ermöglicht.

## Preamble

Throughout the process of cochlear implant (CI) provision, as much information as possible about the technical and physiological function of the implant must be available. In addition, the correct position of the electrode array should be checked regularly. For this purpose, extensive CI-mediated technical and electrophysiological measurements are usually performed intra- and postoperatively [11, 42, 57, 58]. They are an integral part of audiological diagnostics.

Despite international efforts to standardize procedures, there is still great heterogeneity between CI centers in the selection and use of appropriate methods and the associated choice of target parameters [2, 3]. Therefore, the authors believe that there is a need for a standardized process of functional audiological-technical functional testing of the cochlear implant, the determination of the measurement procedures to be performed, and a definition of relevant target parameters. As a result of an open process within the German Working Group for Evoked Response Audiometry (AG-ERA) of the Working Group of German-Speaking Audiologists, Neurotologists, and Otologists (ADANO) of the German Society of Oto-Rhino-Laryngology, Head and Neck Surgery (DGHNO-KHC) and in close cooperation with the Cochlear Implants and Implantable Hearing Systems expert committee of the German Society of Audiology (DGA), a minimum standard protocol for functional performance of audiological-technical functional tests of cochlear implants throughout the care process has been defined and a consensus has been reached.

This minimum standard does not apply only to CI centers but also explicitly to CI companies. It is essential that appropriate measurement and evaluation procedures are provided by the manufacturers and that these procedures are integrated into the respective clinical software solutions. These measures should ensure that the measurements of the defined target parameters can be reliably recorded, reproducibly evaluated, and made comparable between different CI centers.

The use of electrode impedance measurements [1, 29, 30, 37, 40, 41, 55, 57], measurements of the stimulation current-induced non-stimulating electrode voltage [5, 6, 17, 22, 25, 45, 54, 59], electrically evoked stapedius reflexes [8, 16, 31, 53, 56, 57], electrically evoked compound action potentials and auditory brainstem responses [7, 9, 13, 14, 18–21, 26, 27, 31, 32, 34–36, 38, 39, 43, 47, 51, 52, 60], or late cortical auditory evoked potentials [15, 27, 33, 46, 48, 58] and extra- and intracochlear electrocochleography during and after electrode insertion [10, 23, 24, 28] offers a wide range of possible target variables to be measured. In addition, there are various manufacturer-specific options and implementations of the aforementioned measurement methods in the respective clinical software. However, some of these methods are not currently integrated as standard by all manufacturers. Therefore, these recommendations also include information on the respective measurement procedures. On the one hand, the defined target parameters and process recommendations are intended to enable a profound assessment of the measurement results in order to confirm the regular technical and physiological function of the implant. On the other hand, they should contribute to ensure the quality of care [49] in accordance with the currently valid S2k guideline “Cochlea-Implantat Versorgung” of the Association of Scientific Medical Societies (AWMF Registry No.: 017-071; [4]) and the white paper “Cochlea-Implantat (CI)-Versorgung” of the DGHNO-KHC [12] and should also be used for the German CI Register, if applicable [50].

The application of other methods and the selection of additional target parameters for the investigation of individual questions, e.g., in the case of a relative contraindication to CI treatment (see [4, p. 33]), should explicitly not be restricted by this recommendation.

## Recommendations

**Fig. 1:**
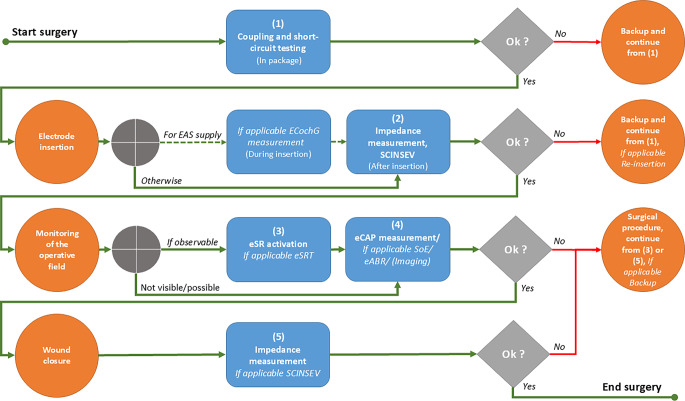
Description of the audiological-technical functional testing of the cochlear implant during surgery: After the coupling and implant short-circuit testing in the package and/or in sodium chloride solution *(1),* the electrode array is inserted, if necessary, with real-time electrocochleography (*ECochG*) monitoring in the case of residual hearing preservation surgery (*EAS*). The electrical (technical) function of the cochlear implant is checked by impedance measurement and measurement of the impedance or voltage matrix (*SCINSEV*) *(2)*. If an electrode problem is detected, the possible cause should be investigated immediately and a replacement implant should be used if necessary. If possible, electrical stimulation of the stapedius reflexes (*eSR*), if necessary, with the determination of the stimulation threshold (*eSRT*) *(3)* and measurement of the electrically evoked compound action potentials (*eCAP*) *(4)* is then performed under observation of the surgical site. In the case of difficult questions or unclear results, electrically evoked brainstem potentials (*eABR*) and/or the spread of excitation (*SoE*) is also measured, and intraoperative imaging is performed if required. If necessary, surgical intervention and/or placement of the backup implant will follow. The impedance measurement is repeated after wound closure *(5)*

If audiological–technical functional tests of the CI or electrophysiological functional tests of the hearing system are carried out during and after electrode insertion in accordance with these recommendations, the methods listed in Table 1 (column 1) should be used for various questions and the specified target parameters (column 3) should be measured, determined, and reported during the surgery in accordance with the process description in Fig. 1. The listed process recommendations (column 4) should also be observed.

**Table 1:** Target parameter and process recommendations for the different measurement methods

Method	Objective	Target parameter recommendation (end points)	Process recommendation	Regular findings (minimum consensus)
***Standard (provision required)***				
Coupling and implant short-circuit test	Testing for bidirectional telemetric data transmission Checking that the implant functions correctly in accordance with the specifications, including the electrode contacts Indications of defects in the implant	Coil-implant coupling [yes/no] Impedances [Z in kΩ]	*Measurement in all available stimulation modes on all electrode contacts of the implant* **pre-OP: **before the start of surgery in the sterile packaging (alternatively: check in NaCl solution directly before insertion) **intra/post-OP: **monitoring of the coupling during the entire audiological-technical diagnostics	**(1) Stable coupling and bidirectional data transmission** **(2) No short circuits between the electrode contacts** [3, 57, 58]
Electrode impedance measurements	Checking that the implant is functioning correctly according to specifications Check all electrode contacts on the electrode array and the external reference electrode(s) Indications of implant failure	Impedances [Z in kΩ] Ground path impedance [Z in kΩ]	*Measurement in all available stimulation modes on all electrode contacts of the implant* **intra-OP: **directly after insertion (if necessary, after electrode fixation) ***and ***after wound closure **post-OP: **regularly (e.g., before each fitting)	**(1) No indication of open circuits (no noticeable impedances)** **(2) No short circuits between the electrode contacts** **(3) Impedances of all intracochlear electrodes within the expected range of the respective implant model or type of electrode array** [1, 29, 30, 37, 40, 41, 55, 57]
Measurements of the stimulation current-induced non-stimulating electrode voltage (SCINSEV)	Checking that the implant is functioning correctly according to specifications Estimation of the electrode position(s) and identification of a defect such as *tip fold-over, base kinking* Research is currently focusing on the insertion depth and position of the electrode array, e.g., for migration of the electrode array [5, 6]	Voltage per current unit [*R*_n,m_ in kΩ]	*Measurement of SCINSEV and visualization in a 12* *×* *12, 16* *×* *16 or 22* *×* *22 matrix (depending on the implant model)* **intra-OP: **after electrode array insertion (if necessary, after electrode array fixation) **post-OP: **regularly in the course of the fitting procedure	**(1) No indication of open circuits (no noticeable impedances)** **(2) No short circuits between the electrode contacts** **(3) Continuous decrease in voltage per unit current at the recording non-stimulating electrode with increasing distance from the stimulating electrode** [17, 22, 25, 45, 54, 59]
Electrically evoked stapedius reflexes (eSR)	Physiological functional testing of the signal transmission (lower auditory pathway—olive complex—brainstem level) and reflex triggering by the facial nerve Confirmation of complete insertion or exclusion of a possible incorrect insertion of the electrode array Determination of the upper limit of the electrical dynamic range (over- or understimulation) Plausibility check of stimulation parameters and eCAP	Reflex triggering [can be evoked/cannot be evoked] eSR thresholds [*Q *in nC]	*Stimulation on several electrode contacts distributed over the electrode array (basal—medial-apical)* **intra-OP**: visual detection (ipsilateral) after initial impedance and measurement of SCINSEV under visual control (intraoperative situs) during electrical stimulation **post-OP**: if required and if feasible, measurement using an impedance audiometer with direct electrical stimulation (ipsilateral) at selected electrodes or determination of the ESR thresholds in the free sound field	**(1) Reflex can be evoked and identified as movement of the staple tendon and/or head** [8, 16, 31, 53, 56, 57]
Electrically evoked compound action potentials of the auditory nerve (eCAP)	Stimulus response threshold diagnostics/threshold profile (initial findings and data for CI fitting) Monitoring of neuronal parameters/retrocochlear diagnostics in the case of unclear integrity of the auditory nerve Position check of the electrode array (e.g., exclusion of *tip fold-over*, electrode migration)	Detectability of the eCAP [yes/no] eCAP thresholds [*Q *in nC] eCAP growth functions (AGF), N1-P1 amplitude [*U *in µV] Absolute latencies of N1 and P1 [t in µs] SoE profile, N1-P1 amplitudes [*U *in µV]	*Determination of eCAP-AGF, eCAP latencies and eCAP thresholds on all intracochlear electrodes (12-22, depending on implant model)* *Measurement of one or more SoE profiles on several electrodes (apical), depending on the implant model and electrode array* **intra-OP: **after impedance measurement, if necessary, also after electrode conditioning, followed by SoE measurement if required **post-OP: **regularly (before/at fitting) after impedance measurement, if required	**(1) eCAP detectable on all electrodes** **(2) eCAP thresholds are within the expected range of the respective implant model (depending on the values of the stimulation parameters)** [7, 19, 26, 31, 32, 36, 38, 47] **(3) In the case of SoE measurement: while maintaining a defined test electrode position (test and recording electrode), the influence of the displacement of the masking position (masking electrode) along the electrode carrier decreases continuously (depending on the values of the stimulation parameters and the respective implant model)** [9, 14, 21, 35, 39, 43]
Electrically evoked auditory brainstem responses (eABR)	Retrocochlear diagnostics for auditory synaptopathy/neuropathy (AS/AN) and unclear integrity of the auditory nerve and lower brainstem, detection of maturation and degeneration processes Assessment of stimulus processing and transmission in special cases, e.g., when eCAP measurement is not possible (in the case of acquired changes to the inner ear, e.g., following chronic inflammation, traumatic changes) Stimulus response threshold diagnostics	Absolute latencies of the waves eIII and eV and inter-peak latency (IPL) as a function of the stimulus intensity [t in ms] Smallest stimulus with detectable and reproducible stimulus response threshold [*Q *in nC] Potential amplitudes [*U *in nV]	*Potential measurement on selected (intracochlear) electrode contacts or areas, detection of the potential latencies and determination of the stimulus threshold* **intra-OP: **if required, measurement via an AEP system after impedance/eSR/eCAP measurement, position of the recording electrodes: Cz or Fpz vs. mastoid or earlobe (contralateral) or in the lower area of the *sternocleidomastoid muscle *(ipsilateral) **post-OP: **if required, measurement via an AEP system, position of the recording electrodes: Cz or Fpz vs. mastoid or earlobe (ipsi- and contralateral)	**(1) Brainstem potentials can be triggered above threshold, waves eIII and eV can be identified, absolute latencies and interpeak latencies are within the expected range (depending on the values of the stimulation parameters)** **(2) eABR thresholds are within the expected range (depending on the values of the stimulation parameters)** [13, 18, 20, 27, 34, 51, 52, 58, 60]
*For EAS/hybrid (provision useful in individual cases)*				
Electrocochleography (ECochG)	Control and monitoring of the cochlear function of hair cells and afferent auditory nerve fibers (residual auditory function) before, during, and after insertion of the electrode array	Detectability of the 3 signal components of the ECochG (CM, CAP, SP), at least “cochlear microphonic” (CM) Determination of the (largest) CM amplitude [*U *in µV]	*During insertion: Acoustic stimulation, e.g., at 500* *Hz (tone burst) with approx. 40* *dB SL and potential measurement on selected electrode contacts* **intra-OP: **if required recording of the ECochG *extracochlear: *electrode on the round window (before insertion) or on the promontory (during insertion), * intracochlear: *apical electrode; in each case in reference to a lead electrode (Cz or Fpz) **post-OP: **if required, recording of the ECochG *intracochlear: *apical electrode (22 or 1 depending on implant model); in each case in reference to a lead electrode (Cz or Fpz)	**No (rapid) drop in amplitude in the ECochG during insertion of the electrode carrier** **Intra- and post-OP stable CM amplitudes** [10, 23, 24, 28]
*Optional (mainly of scientific interest)*				
Electrically evoked auditory cortical responses (eACR)	Integrity testing of the auditory pathway up to the level of the cerebral cortex Assessment of auditory pathway function and maturation at cortical level	P1-N1-P2-N2 complex [t in ms]	*Potential measurement on selected electrode contacts or areas, detection of the potential latencies and determination of the stimulus response threshold* **post-OP: **if required, measurement via an AEP system, position of the recording electrodes: Cz or Fpz vs. mastoid or earlobe (ipsi- and contralateral)	**(1) Cortical potentials can be triggered above threshold, P1-N1-P2-N2 complex well detectable, absolute latencies and interpeak latencies are within the expected range (depending on the values of the stimulation parameters)** [15, 27, 33, 46, 48, 58]

*AEP* auditory evoked potentials, *AGF* amplitude growth function, *CAP* compound action potential, *CI* cochlear implant, *CM* cochlear microphonic, *Cz* electrode positioned at vertex, *eABR* electrically evoked auditory brainstem responses , *eACR* electrically evoked auditory cortical responses, *EAS* electrically acoustic stimulation, *eCAP* electrically evoked compound action potential, *ECochG* electrocochleography, *eSR* electrically evoked stapedius reflex, *eSRT* electrically evoked stapedius reflex threshold, *Fpz* frontocentral placement of electrode, *NaCl* sodium chloride, *SCINSEV* stimulation current-induced non-stimulating electrode voltage, *SL* sensation level, *SoE* spread of excitation, *SP* summation potential

The highest possible quality of the examination results is ensured by guaranteeing the functional integrity of all medical device components, creating optimal measurement conditions (appropriate examination room) by reducing artifacts at the lowest possible noise levels and/or eliminating/shielding electromagnetic interference fields, minimizing residual noise and residual interference (e.g., by relaxing/sleeping/sedating/anesthetizing the person to be examined), and the professional qualifications and experience of the operators.

## Definitions

### Stimulation current-induced non-stimulating electrode voltage.

Indirect measure of the electric field propagation along the electrode array and out of the *cochlea *(in the text also abbreviated as SCINSEV [44]), measured as voltage per unit of current and represented in an *m* × *n* matrix*, *i.e., the electric voltage *U *induced by the input current *I *at a stimulation electrode *(m) *as the potential difference between a (non-stimulating) recording electrode (*n*) and a ground electrode, expressed in the unit of measurement kΩ with

$$\boldsymbol{R}_{m,n}=\frac{\boldsymbol{U}_{n}}{\boldsymbol{I}_{m}}.$$

### Stimulus response threshold.

The lowest applied electrical charge *Q *with (still) identifiable and reproducible stimulus response (also referred to as “threshold” in Table 1) as the product of stimulation current and stimulation time (pulse phase time), specified in the unit of measurement nC with

$$\boldsymbol{Q}=\boldsymbol{I}\cdot \boldsymbol{t}.$$

If this unit of measurement is not available, the manufacturer-dependent intensity unit should be specified and labeled accordingly.

## Declarations

For this article no studies with human participants or animals were performed by any of the authors. All studies followed the ethical standards indicated in each case.

The supplement containing this article is not sponsored by the industry.
